# Glucose oxidase as an alternative to antibiotic growth promoters improves the immunity function, antioxidative status, and cecal microbiota environment in white-feathered broilers

**DOI:** 10.3389/fmicb.2023.1100465

**Published:** 2023-03-03

**Authors:** Wenyu Zhao, Yuan Huang, Na Cui, Ruiguo Wang, Zhiming Xiao, Xiaoou Su

**Affiliations:** Key Laboratory of Agro-Product Quality and Safety of the Ministry of Agriculture, Institute of Quality Standards and Testing Technology for Agro-Products, Chinese Academy of Agricultural Sciences, Beijing, China

**Keywords:** glucose oxidase, broiler, antibiotic, healthy condition, microbiota

## Abstract

This study aimed to demonstrate the effects of glucose oxidase (GOD) on broilers as a potential antibiotic substitute. A total of four hundred twenty 1-day-old male Cobb500 broilers were randomly assigned into five dietary treatments, each with six replicates (12 chicks per replicate). The treatments included two control groups (a basal diet and a basal diet with 50 mg/kg aureomycin) and three GOD-additive groups involving three different concentrations of GOD. Analysis after the *t*-test showed that, on day 21, the feed:gain ratio significantly decreased in the 1,200 U/kg GOD-supplied group (GOD1200) compared to the antibiotic group (Ant). The same effect was also observed in GOD1200 during days 22–42 and in the 600 U/kg GOD-supplied group (GOD600) when compared to the control group (Ctr). The serum tests indicated that, on day 21, the TGF-β cytokine was significantly decreased in both GOD600 and GOD1200 when compared with Ctr. A decrease in malondialdehyde and an increase in superoxide dismutase in GOD1200 were observed, which is similar to the effects seen in Ant. On day 42, the D-lactate and glutathione peroxidase activity changed remarkably in GOD1200 and surpassed Ant. Furthermore, GOD upregulated the expression of the jejunal barrier genes (MUC-2 and ZO-1) in two phases relative to Ctr. In the aureomycin-supplied group, the secretory immunoglobulin A significantly decreased in the jejunum at 42 days. Changes in microbial genera were also discovered in the cecum by sequencing 16S rRNA genes at 42 days. The biomarkers for GOD supplementation were identified as *Colidextribacter, Oscillibacter, Flavonifractor, Oscillospira*, and *Shuttleworthia*. Except for *Shuttleworthia*, all the abovementioned genera were n-butyrate producers known for imparting their various benefits to broilers. The PICRUSt prediction of microbial communities revealed 11 pathways that were enriched in both the control and GOD-supplied groups. GOD1200 accounted for an increased number of metabolic pathways, demonstrating their potential in aiding nutrient absorption and digestion. In conclusion, a diet containing GOD can be beneficial to broiler health, particularly at a GOD concentration of 1,200 U/kg. The improved feed conversion ratio, immunity, antioxidative capacity, and intestinal condition demonstrated that GOD could be a valuable alternative to antibiotics in broiler breeding.

## Introduction

Antibiotics have been playing a vital role in commercial poultry production since their first use in the 1940s (Castanon, 2007). They greatly improve the production efficiency of the breeding industry and satisfy the increasing human demand for animal-derived foods. However, their continued use has had negative impacts. The issue of antibiotic resistance has attracted a great amount of attention because of the existing and potential threats antibiotics pose to public health (Laxminarayan et al., 2014). Many countries, such as those in the European Union and the USA, started to restrict or even forbid the use of antibiotics in industrial-scale animal production (Marshall and Levy, 2011). From 1 January 2020, the Ministry of Agriculture of China also issued regulations prohibiting the use of any growth-promoting antibiotics. Thus, it is essential to find effective in-feed antibiotic alternatives in animal production without compromising human health. Accordingly, novel products, including plant essential oils (Brenes and Roura, 2010), probiotics (Kabir et al., 2005), organic acids (Adil et al., 2010), antibacterial peptides (Wang et al., 2016), and feed enzymes (Askelson et al., 2018), were investigated.

Glucose oxidase (GOD), as one of the feed enzymes, could specifically catalyze the oxidation of β-D-glucose to gluconic acid and hydrogen peroxide (Bankar et al., 2009).

This enzyme has been gradually accepted by the feed industry due to several verified advantages, including growth promotion, feed quality improvement, intestinal health regulation, and toxic reaction reduction, with non-toxic, low-residue characteristics (Dang et al., 2021, 2022; Hoque et al., 2022; Sun et al., 2022). In animals, particularly poultry, intestinal barrier and microbiota compositions are critical, since they are closely related to the immune system and health (Robinson et al., 2015; Awad et al., 2017; Pandit et al., 2018). The gastrointestinal tract's microbiota flora is linked to “intestinal” or “non-intestinal” functions ranging from nutrient absorption to immune response and even the gut–brain axis (Gao et al., 2017; Borda-Molina et al., 2018). Therefore, animal nutrition research mainly focuses on the host gut which correlates with optimal health and productivity. GOD has been known to help animals avoid intestinal dysfunction or other gut problems based on its reaction mechanism (Qu and Liu, 2021). The GOD-catalyzed glucose products can act on the gut of broilers, gluconic acid can produce the short-chain fatty acids (SCFAs) and further create a weakly acidic intestinal tract environment (Mortensen et al., 1988; Biagi et al., 2006), and hydrogen peroxide can participate in the oxidative stress response and regulate gut microbiota through its bactericidal and antimicrobial properties (Vatansever et al., 2013; Belambri et al., 2018). Though some researchers recently elucidated the effects of GOD with a sequencing-based technique (Wu et al., 2020; Meng et al., 2021), it is still ambiguous how GOD improves gut health and immunity function and why antibiotics can be replaced by it in broiler production (Liang et al., 2022). Some voids, such as how the additive, defense function, and growth performance interact with each other, still remain.

Therefore, this study aimed to determine the impact of glucose oxidase on the growth performance, immunity, antioxidative stage, and intestinal function of white-feathered broilers and attempted to explain it from the perspective of intestinal microorganisms. These findings may contribute to expanding the knowledge concerning the application of glucose oxidase. Furthermore, the comparison between the GOD and aureomycin-supplemented groups can further illustrate the role of GOD in feed as a substitute for antibiotic growth promoters (AGPs).

## Materials and methods

### Birds, diet, and management

A total of four hundred twenty 1-day-old male Cobb500 white-feathered broiler chicks obtained from Beijing Poultry Breeding Co., Ltd. were randomly assigned into five dietary treatments, each in six replicates (12 chicks/replicate) by cage-rearing, and the original average weight of every replicate had no remarkable difference. The control group (Ctr) was fed with a basal diet formulated to meet the nutrient requirements of poultry as per the National Research Council 1994, and other treatment groups were based on the basal diet with the addition of various feed additives. The antibiotic group (Ant) was supplied with 50 mg/kg aureomycin (Chia Tai Co., Ltd., Henan, China). Different concentrations of GODs (300, 600, and 1,200 U per kilogram of diet) were determined from the doses recommended by the manufacturer (VTR Bio-tech Co., Ltd., Zhuhai, Guangdong, China) and from massive references for their effective applications in the poultry industry, and named as GOD300, GOD600, and GOD1200. Table 1 details the diet compositions and nutrient contents of the basal diet for the entire study's starting (day 0–21) and growing (day 21–42) phases. All the chickens were exposed to incandescent light for a 24-h photoperiod instead of daylight, and the birds were allowed *ad libitum* access to drinking water from nipple drinkers. The diets for the chickens were mash feed for the first 12 days and then gradually transited to pellet diets. For temperature, ventilation, and other types of ventilation management for the birds in this research, one is referred to the guidelines for raising meat-type broilers (National Technical Committee for Animal Agriculture Standardization, 2005). Feed consumption and body weight were recorded every week and the mortality of the birds was checked daily. These data were used to calculate the feed intake, body weight gain, and feed conversion ratio.

**Table 1:** Composition and nutrient levels of basal diet in the two phases of trial (% as fed basis).

**Items**	**Contents (%)**	
	**1–21 d of age**	**22–42 d of age**
**Ingredients**		
Corn	56.59	59.96
Soybean meal	25.95	20.00
Cottonseed meal	4.50	4.42
Corn gluten meal	4.00	5.00
Wheat middling	2.00	2.00
Soybean oil	2.49	4.50
Calcium hydrogen phosphate	1.82	1.58
Limestone	1.35	1.27
Salt	0.35	0.35
*L*-Lysine-HCl	0.35	0.35
*DL*-Methionine	0.23	0.21
Threonine	0.05	0.04
Mineral Premix^a^	0.20	0.20
Vitamin Premix^b^	0.02	0.02
Choline chloride	0.10	0.10
Total	100.00	100.00
**Nutrients**		
ME (kcal/kg)	2,980.00	3,160.00
Crude protein	21.95	19.95
Ca	1.00	0.90
Non-phytate P	0.45	0.40
Lysine	1.30	1.15
Methionine	0.58	0.54
Methionine + cystine	0.94	0.87
Threonine	0.84	0.75
Tryptophan	0.23	0.20

a The Mineral Premix supplied the following (per kilogram of complete feed): Cu, 8 mg; Zn, 75 mg; Fe, 80 mg; Mn, 100 mg; I, 0.35 mg; and Se, 0.15 mg.

b The Vitamin Premix supplied the following (per kilogram of complete feed): vitamin A, 12,500 IU, vitamin D_3_, 2,500 IU, vitamin E, 18.75 mg, vitamin K_3_, 2.65 mg, vitamin B_1_, 2 mg, vitamin B_2_, 6 mg, vitamin B_12_, 0.025 mg, biotin, 0.0325 mg, folic acid, 1.25 mg, pantothenic acid, 12 mg, and niacin, 50 mg.

### Sample collection

One chicken with an average weight from each replicate was chosen for sample collection after a 12-h fast at the end of the two phases (days 21 and 42). The blood samples were drawn from the wing vein and dropped into tubes without anticoagulants. The serum used in further research was received after the blood samples were centrifuged at 3,500 × g for 10 min (4°C) and stored at −80°C. Birds were killed and shortly dissected after collecting blood samples. Immune organs (the thymus, the spleen, and the bursa of Fabricius) were taken out from the dead body individually, following the rinsing, blotting, and weighting procedures. The segments (~2 cm) in the middle of the jejunum were collected, washed with physiological saline, and then dropped into 10% neutral-buffered formalin for immobilization. Meanwhile, the remaining segments of the jejunum were gently scraped to sample the mucous membrane, snap-frozen in liquid nitrogen, and stored at −80°C for gene expression analysis. Then, under the condition of being germ-free, the cecal part of the bird was gathered. Its contents were speedily squeezed into sterile cryopreservation tubes and then stored in liquid nitrogen as described previously.

### Biochemical index and enzyme activity analysis

Biochemical index and enzyme activity were measured after the collected, frozen serum samples finished the two-step gradient thawing. Alanine aminotransferase (ALT), aspartate transaminase (AST), total protein (TP), alkaline phosphatase (ALP), and urea were all determined by a Cobas 6000 automatic biochemical analyzer (Roche Diagnostics Co., Ltd., Shanghai, China). The enzyme activities of glutathione peroxidase (GSH-Px), diamine oxidase (DAO), and also the malonaldehyde (MDA) concentration of serum were measured by colorimetric methods with a T9CS+ spectrophotometer (Purkinje General Instrument Co., Ltd., Beijing, China). A microplate reader detected the total antioxidant capacity (T-AOC) and superoxide dismutase (SOD). All the antioxidant indexes aforementioned were conducted according to the manufacturer's instructions.

### ELISA

Transforming growth factor-β (TGF-β), D-lactate (D-Lac), diamine oxidase (DAO), and 8-hydroxy-2′-deoxyguanosine (8-OH-dG) in the serum were measured through enzyme-linked immunosorbent assay kits (Nanjing Jiancheng Institute of Bioengineering, Nanjing, China).

### Immunohistochemical observations of jejunal secretory immunoglobulin A

Jejunum samples fixed in 10% neutral-buffered formalin for over 24 h were embedded in paraffin. The 4-μm tissue slices were prepared by Leica RM2255 (Leica Biosystems, Wetzlar, Germany). Afterward, dewaxing and dehydration of the samples were executed and 3% H_2_O_2_ was used to remove the endogenous peroxidase activity in slices. Next, the primary antibody (SouthernBiotech, Birmingham, AL, USA) and secondary antibody (Thermo Fisher Scientific, Fremont, CA, USA) were applied for incubation of the samples accordingly, the former left overnight at 4°C and the latter for 10 min at room temperature, along with the color reaction visualized by the DAB chromogen. The SIgA-positive cells were stained prominently brown in contrast to the surrounding tissue, which was counterstained for identifying host cells. Finally, the slides were observed under the microscope (Olympus Corporation, Tokyo, Japan).

### Total RNA extraction and gene expression in the jejunum

The total RNA was extracted from collected jejunal mucosa using the RNA Easy Fast Tissue Kit (Tiangen Biotech Co., Ltd., Beijing, China) following the standard operating procedure. Nanodrop 2000 spectrophotometer (Thermo Fisher Scientific, Waltham, MA, USA) and agarose–ethidium bromide electrophoresis were applied to determine the concentration, purity, and integrity of the RNA. The synthesis of complementary DNA (cDNA) and further real-time PCRs in duplicate were all performed with the One Step TB Green^®^ PrimeScript^TM^ RT-PCR Kit II (TaKaRa, Dalian, China) and the ABI 7500 Fast Real-Time PCR system (Applied Biosystems, Waltham, MA, USA). The information on primer sequences of *Claudin-1, Occludin, ZO-1, MUC-2*, and β*-actin* is given in Table 2. The eukaryotic reference gene β*-actin* was used to normalize the relative gene quantification by the 2^−ΔΔCt^ method (Livak and Schmittgen, 2001).

**Table 2:** Sequences of target and reference genes used for the evaluation of intestinal function.

**Gene name**	**Primers (5′** **to 3** **′** **)**	
	**Forward**	**Reverse**
*ZO-1*	CTTCAGGTGTTTCTCTTCCTCCTC	CTGTGGTTTCATGGCTGGATC
*Occludin*	GCAGATGTCCAGCGGTTACTAC	CGAAGAAGCAGATGAGGCAGAG
*Claudin-1*	ACAACATCGTGACGGCCCA	CCCGTCACAGCAACAAACAC
*MUC-2*	AGGAATGGGCTGCAAGAGAC	GTGACATCAGGGCACACAGA
*β-actin*	GAGAAATTGTGCGTGACATCA	CCTGAACCTCTCATTGCCA

*ZO-1*, Zona occludens 1; *MUC-2*, Mucin 2.

### 16srRNA amplification and illumina sequences

The cecal contents at the end of 42 day were extracted using the E.Z.N.A.^®^ Stool DNA Kit (Omega Bio-tek, Norcross, GA, USA) for microbial DNA as per the manufacturer's instructions. The DNA extract's quality was checked similarly to that of RNA. Hypervariable region V3–V4 of the bacterial 16S rRNA gene was amplified by the primer pair 338F (5′-ACTCCTACGGGAGGCAGCAG-3′) and 806R (5′-GGACTACHVGGGTWTCTAAT-3′) with an ABI GeneAmp^®^ 9700 PCR thermocycler (Applied Biosystems, Waltham, MA, USA). The specific program for the PCR amplification of 16srRNA was conducted as follows: denaturation at 95°C for 3 min, then followed by 27 cycles at 95°C for 30 s, annealing at 55°C for 30 s, then extension at 72°C for 45 s, and a final extension at 72°C for 10 min, and the final temperature was 4°C. The PCR was conducted in triplicate with a 20-μL mixture for one. The mixture was composed of 4 μL of 5 × FastPfu buffer, 2 μL of 2.5 mM dNTPs, 0.8 μL each of forward and reverse primers (5 μM), 0.4 μL of FastPfu DNA Polymerase, 10 ng of template DNA, and the ddH_2_O. Two percent agarose gel and the AxyPrep DNA Gel Extraction Kit (Axygen Biosciences, Union City, CA, USA) were used to finish the extraction and purification of the PCR product, and they were then quantified by a Quantus™ Fluorometer (Promega, Madison, WI, USA).

Purified amplicons were pooled in equal amounts and paired-end sequenced (2 × 300 bp). All the analysis was finished by Majorbio Bio-Pharm Technology Co., Ltd. (Shanghai, China) on the Illumina MiSeq platform (Illumina, San Diego, CA, USA) in accordance with the standard protocol. Finally, the raw reads were deposited into the NCBI Sequence Read Archive (SRA) database (Accession Number: SRP392472).

The in-house Perl script was used to demultiplex the raw FASTQ files that were quality-filtered by fastp version 0.19.6 and merged by flash version 1.2.7 with the following criteria later:

(i) The bases whose quality value is below 20 bp at the end of the reads are filtered. A 50-bp window is set and the back-end bases under 20 of the average quality value are cut off, the reads containing *N* bases are filtered, and the quality control value should be below 50 bp; (ii) paired reads are merged into one sequence with the relationship of overlap between the PE reads. Furthermore, the length of the overlap should be longer than 10 bp; (iii) the overlap region of the spliced sequence with the allowable mismatch rate is screened out to be higher than 0.2; and (iv) the barcode and primers at both ends of the sequence are used to distinguish the samples and adjust their direction. The barcode should have no mismatched primers, and the maximum primer mismatch number is 2.

### Statistical analysis

The data were analyzed by SPSS 25.0 (SPSS Inc., Chicago, IL, USA). The Shapiro–Wilk test was initially used to assess the normality of data. The differences between samples were evaluated using the one-way analysis of variance (ANOVA) and Duncan's multiple comparisons test. Each control group was pairwise compared with GOD300, GOD600, and GOD1200 using the *t*-tests to assess the growth performance indexes. A tendency toward significance was considered at 0.05 ≤ *P* < 0.1, and the statistical significance was stated based on the value of *P* of < 0.05 (Granato et al., 2014).

For microbiota profiling, the processed effective reads were clustered into operational taxonomic units (OTUs) using UPARSE 7.1 (http://drive5.com/uparse/) with 97% sequence similarity. Each 16S rRNA gene sequence was analyzed by the RDP Classifier algorithm (http://rdp.cme.msu.edu/) at different taxonomic levels and then against the Silva (SSU128) 16S rRNA database using a confidence threshold of 70%.

Rarefaction curves and α-diversity indices were calculated by Mothur v1.30.1 (Schloss et al., 2009). The similarity among the microbial communities in different samples was determined by the principal coordinate analysis (PCoA) based on Bray–Curtis dissimilarity using the Vegan v2.5-3 package. The species composition was obtained based on the taxonomic analysis. Analysis of similarities (ANOSIM) was applied to assess the significance of the microbial community differences among various treatments. The Kruskal–Wallis H test and the Wilcoxon rank-sum test were employed to explore the differences in the relative abundance of bacteria among multiple groups and then between every two groups, respectively.

Each OUT representative sequence's taxonomy level was analyzed by an RDP Classifier version 2.2 (Wang et al., 2007) using the confidence threshold of 0.7. The PICRUSt2 (Phylogenetic Investigation of Communities by Reconstruction of Unobserved States) software was used to predict the microbiome function, based on these OUT sequences. All the data aforementioned were analyzed on the platform of Majorbio I-Sanger Cloud Platform (www.i-sanger.com).

## Results

### Growth performance and the immune organ indexes

There were no significant differences in the growth performance indexes after multiple comparisons in five treatments. The specific indexes, including average daily feed intake (ADFI), average daily-weight gain (ADG), and feed:gain ratio (F:G), are shown in Table 3. Furthermore, the *t*-test result in Supplementary Figure S1 indicated that, during 0–21 days, the antibiotic supplement showed a significantly higher ADFI value (*P* < 0.05) and an F:G value (*P* < 0.05) than GOD1200. For days 22–42, the F:G showed a significant increase in the basal diet group when compared to GOD600 (*P* < 0.05) or GOD1200 (*P* < 0.05). Additionally, GOD and the antibiotic-supplemented groups have no significant effect (*P* > 0.05) on the immune organ indexes (organ weight:body weight) during the experimental periods (Table 4).

**Table 3:** Effect of GOD on the growth performance of broilers (mean ± SEM, *n* = 6).

**Variable**	**Ctr**	**Ant**	**GOD300**	**GOD600**	**GOD1200**	**SEM**	***P*-value**
**1–21 d**							
ADFI (g/d)	52.83	52.65	51.65	52.95	51.73	0.773	0.501
ADG (g/d)	39.53	39.27	39.65	39.26	40.60	0.252	0.410
F:G (g:g)	1.34	1.35	1.30	1.35	1.27	0.025	0.444
**22–42 d**							
ADFI (g/d)	128.77	122.76	123.94	121.09	125.98	1.871	0.762
ADG (g/d)	66.06	67.68	65.74	67.38	67.85	0.933	0.944
F:G (g:g)	1.95	1.82	1.89	1.80	1.86	0.022	0.136
**1–42 d**							
ADFI (g/d)	88.80	87.47	88.25	86.07	89.37	1.121	0.910
ADG (g/d)	52.55	54.59	53.00	51.44	53.48	0.771	0.793
F:G (g:g)	1.69	1.62	1.67	1.68	1.66	0.123	0.432

ADFI, feed intake; ADG, body weight gain; F:G, feed:gain ratio; Ctr, control group; Ant, aureomycin group; GOD300, GOD600, and GOD1200 groups represent different concentrations of GOD (300, 600, and 1,200 U/kg).

**Table 4:** Effect of GOD on relative weights of immune organs of broilers (mean ± SEM, *n* = 6).

**Variable**	**Ctr**	**Ant**	**GOD300**	**GOD600**	**GOD1200**	**SEM**	***P*-value**
**Immune index**							
**d 21**							
Spleen	0.66	0.72	0.67	0.69	0.74	0.021	0.761
Thymus	6.03	5.41	4.97	5.03	5.03	0.231	0.570
Bursa of fabricius	1.60	1.37	1.33	1.44	1.53	0.050	0.481
**d 42**							
Spleen	0.89	0.77	0.75	0.80	0.86	0.042	0.852
Thymus	3.03	2.82	3.13	3.26	2.92	0.113	0.743
Bursa of fabricius	1.18	1.27	1.18	1.07	1.229	0.042	0.621

Ctr, control group; Ant, aureomycin group; GOD300, GOD600, and GOD1200 groups represent different concentrations of GOD (300, 600, and 1,200 U/kg).

### Biochemical, cytokine, and antioxidant parameters in serum

The relevant parameters tested in serum are shown in Table 5. For biochemical parameters, ALP was significantly higher in GOD300 than in Ctr (*P* < 0.05) on day 21. However, no effect was observed for ALT, AST, TP, and urea (*P* > 0.1). For cytokines, the GOD supplementation gave rise to significant differences in TGF-β in its moderate and higher dosage groups (*P* < 0.05) on day 21, and the effect was also exerted in the indicator of D-Lac in GOD1200 on day 42 (*P* < 0.05) (Table 6). There were no significant differences in the activity of DAO in both stages. For the antioxidant parameters (Table 7), GOD1200 significantly increased the SOD activity (*P* < 0.05) and showed a trend toward a lower level of MDA content (0.05 ≤ *P* < 0.1) compared with Ctr on day 21. Moreover, it was noted that the GSH-Px activity was extremely significant (*P* < 0.01) at the growth anaphase of broiler in GOD1200, and no differences were found among other GOD treatment groups and the two control groups (*P* > 0.05).

**Table 5:** Effect of GOD on the biochemical parameters of broilers in serum (mean ± SEM, *n* = 6).

**Variable**	**Ctr**	**Ant**	**GOD300**	**GOD600**	**GOD1200**	**SEM**	***P*-value**
**d 21**							
ALT (U/L)	5.00	7.29	6.57	5.00	5.71	0.361	0.194
AST (U/L)	218.00	251.43	252.00	221.29	239.71	5.086	0.112
TP g/dL	2.50	2.43	2.53	2.36	2.40	0.044	0.747
ALP (10^3^ U/L)	13.52^a^	13.26^a, b^	8.84^b^	12.74^a, b^	11.75^a, b^	65.042	0.041
UREA mg/dL	3.00	3.29	3.71	3.43	3.57	0.100	0.227
**d 42**							
ALT (U/L)	< 5.00	< 0.00	< 5.00	< 5.00	< 5.00	–	–
AST (U/L)	323.00	350.43	378.43	295.57	482.86	21.680	0.131
TP g/dL	2.49	2.66	2.56	2.54	2.74	0.055	0.543
ALP (10^3^ U/L)	6.61	3.29	4.24	4.67	4.31	555.062	0.433
UREA mg/dL	2.14	2.43	2.14	2.00	2.00	0.437	0.347

a, b Mean values within a row with no common superscript differ significantly (*P* < 0.05).

ALT, alanine aminotransferase; AST, aspartate transaminase; TP, total protein; ALP, alkaline phosphatase; Ctr, control group; Ant, aureomycin group; GOD300, GOD600, and GOD1200 groups represent different concentrations of GOD (300, 600, and 1,200 U/kg).

**Table 6:** Effect of GOD on cytokine parameters and serum markers of broilers in serum (mean ± SEM, *n* = 6).

**Variable**	**Ctr**	**Ant**	**GOD300**	**GOD600**	**GOD1200**	**SEM**	***P*-value**
**d 21**							
TGF-β (ng/L)	321.76^a^	271.34^a, b^	197.14^a, b^	173.56^b^	154.18^b^	20.891	0.062
**Serum enterotoxin markers**							
D-Lac (nmol/mL)	6.56	4.00	6.90	6.05	6.24	0.900	0.872
DAO (U/L)	17.37	18.85	19.61	15.22	14.45	0.841	0.201
**d 42**							
TGF-β (ng/L)	203.76	226.80	203.17	202.88	199.87	13.084	0.971
**Serum enterotoxin markers**							
D-Lac (nmol/mL)	27.58^a^	30.64^a^	28.39^a^	28.55^a^	5.94^b^	3.002	0.031
DAO (U/L)	24.23	25.84	24.06	23.54	18.74	1.046	0.231

a, b Means within a row with no common superscripts are significantly different (*P* < 0.05).

TGF-β, transforming growth factor-β; D-Lac, D-lactic acid; DAO, diamine oxidase; Ctr, control group; Ant, aureomycin group; GOD300, GOD600, and GOD1200 groups represent different concentrations of GOD (300, 600, and 1,200 U/kg).

**Table 7:** Antioxidant parameters in the serum of broilers (mean ± SEM, *n* = 6).

**Variable**	**Ctr**	**Ant**	**GOD300**	**GOD600**	**GOD1200**	**SEM**	***P*-value**
**d 21**							
T-AOC (U/L)	1.17	1.21	1.23	1.23	1.26	0.033	0.932
SOD (U/ml)	108.57^a^	113.89^a, b^	110.83^a, b^	105.30^a^	119.92^b^	1.612	0.041
GSH-Px (U/ml)	486.39	505.97	527.71	520.53	565.58	14.471	0.573
8-OH-dG (ng/mL)	25.22	29.21	25.17	27.41	25.99	1.056	0.721
MDA (nmol/ml)	3.17^a^	2.31^b^	2.83^a, b^	2.79^a, b^	2.61^b^	0.100	0.090
**d 42**							
T-AOC (U/L)	0.67	0.72	0.76	0.75	0.77	0.031	0.795
SOD (U/ml)	123.19	127.45	123.42	124.13	128.12	1.072	0.467
GSH-Px (U/ml)	1,516.27^A^	1,353.97^A^	1,742.03^A^	1,693.47^A^	2,231.39^B^	87.153	< 0.01
8-OH-dG (ng/mL)	55.88	61.28	54.13	53.68	55.58	1.138	0.221
MDA (nmol/ml)	1.63	1.55	1.63	1.62	1.62	0.036	0.873

a, b Means within a row with no common superscripts are significantly different (*P* < 0.05) or have a tendency toward significance (0.05 < *P* < 0.1).

A, B Means within a row with no common superscripts are extremely significantly different (*P* < 0.01).

T-AOC, total antioxidative capacity; SOD, superoxide dismutase; GSH-Px, glutathione peroxidase; MDA, malondialdehyde; 8-OH-dG, 8-hydroxy-2′-deoxyguanosine; Ctr, control group; Ant, aureomycin group; GOD300, GOD600, and GOD1200 groups represent different concentrations of GOD (300, 600, and 1,200 U/kg).

### Jejunal secretory immunoglobulin A

**Figure 1:**
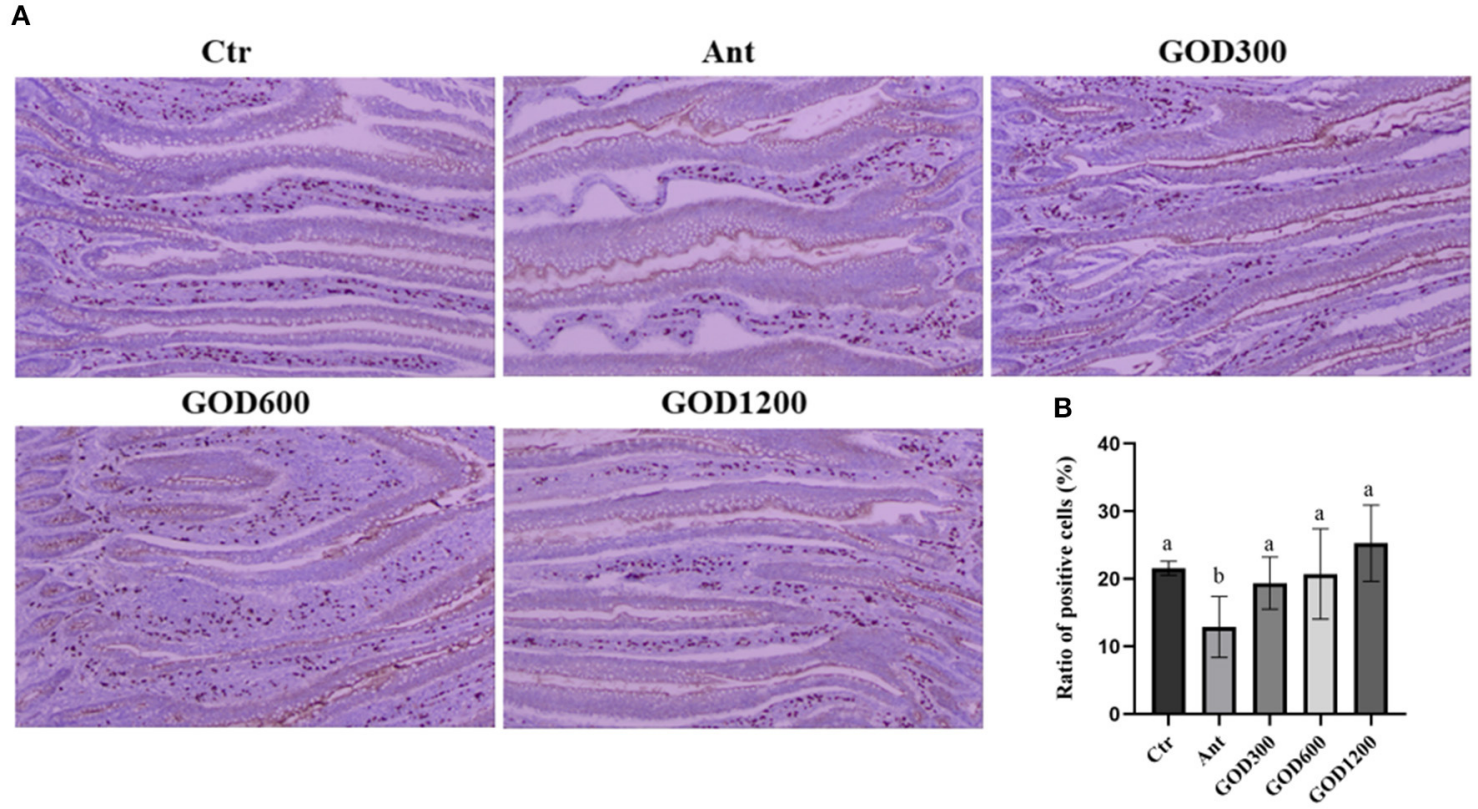
The effect of GOD-supplemented dietary on SIgA distribution in the jejunum of broilers on day 42 was examined by immunohistochemistry. SIgA-positive cells were stained prominently brown. The pictures were observed at 100× magnification (*n* = 6) **(A)**. The ratio of positive cells was calculated and analyzed among the five treatment groups **(B)** (*P* < 0.05). Ctr, negative control fed with the basal diets; Ant, positive control fed with the basal diets added 50 mg/kg aureomycin; GOD300, GOD600, and GOD1200, the basal diets supplied with 300, 600, and 1,200 U/kg glucose oxidase, respectively. ^a,b^Means within a row with no common superscripts are significantly different (*P* < 0.05). Ctr, negative control group fed with the basal diets; Ant, positive control group fed with the basal diets added 50 mg/kg aureomycin.

The distribution of SIgA in the jejunum and the proportion of positive cell ratio on day 42 are given in Figure 1. The SIgA-positive cells were prominently stained brown compared with the surrounding tissues. Although no significant differences were observed in the basal diet and GOD-supplied groups, the positive cell ratio in aureomycin-supplied group was remarkably decreased among the whole treatments (*P* < 0.05).

### Gene expressions in the jejunum related to intestinal tight junctions

**Figure 2:**
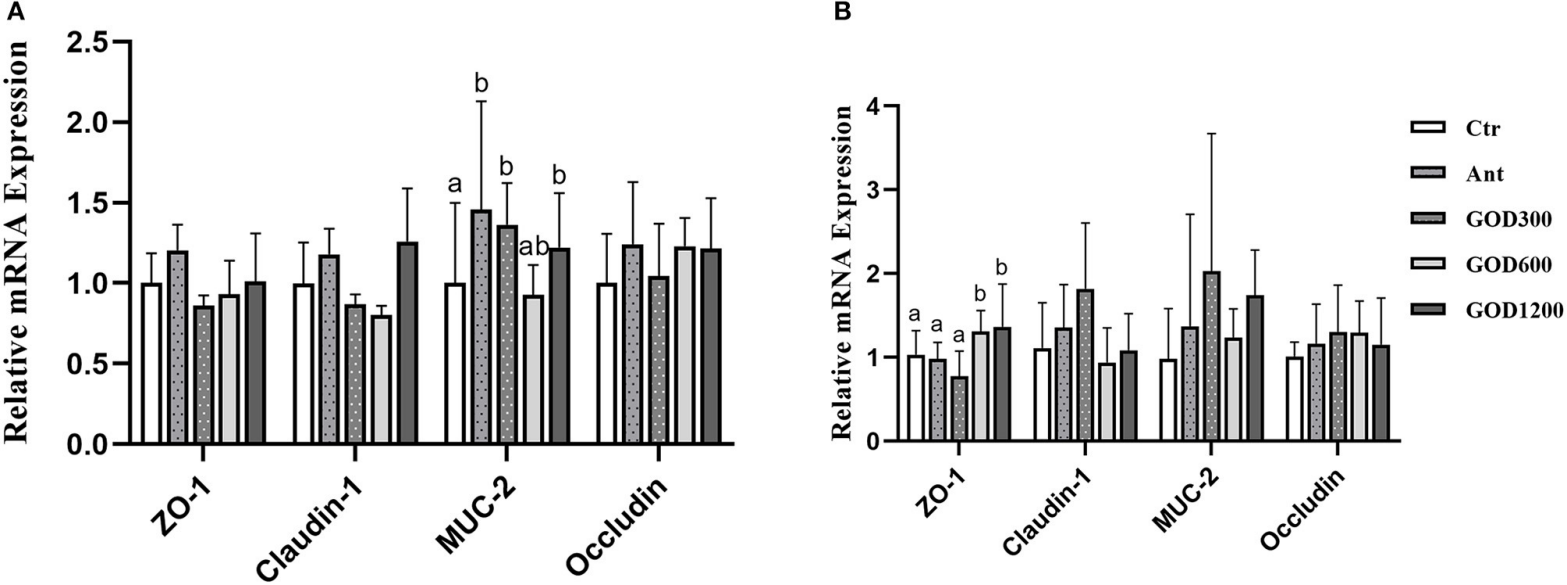
The effect of GOD-supplemented dietary on the relative mRNA expression of jejunal junction protein genes and the mucin gene of broilers during both days 0–21 **(A)** and 22–42 **(B)** growth phases. ^a,b^Means within a row with no common superscripts are significantly different (*n* = 6, *P* < 0.05). Ctr, negative control fed with the basal diets; Ant, positive control fed with the basal diets added 50 mg/kg aureomycin; GOD300, GOD600, and GOD1200, the basal diets supplied with 300, 600, and 1,200 U/kg glucose oxidase, respectively. ^a,b^Means within a row with no common superscripts are significantly different (*P* < 0.05). Ctr, negative control group fed with the basal diets; Ant, positive control group fed with the basal diets added 50 mg/kg aureomycin.

Figure 2A shows that, compared with Ctr, GOD300, and GOD1200 showed a significant increase in the content of *Mucin-2* (*MUC-2*) (*P* < 0.05) and the effect was the same as that of Ant during the first growth stage of broilers (0–21 days). There were no notable differences in the extra three jejunal junction protein genes (*ZO-1, Claudin-1*, and *Occludin*) during this period (*P* > 0.05). During the late growth stage (Figure 2B), we found that the relative mRNA expression of *ZO-1* upregulated apparently in GOD600 and GOD1200 compared with Ctr or Ant (*P* < 0.05). However, no other significant differences were found in the expression of *Claudin-1, MUC-2*, and *Occludin*.

### Microbiota analysis by 16SrDNA

After filtering, an average of 50,736 reads per sample was obtained. The rarefaction curves are plotted in Supplementary Figure S2 to provide the complete evidence of adequate sequencing depth. As the result showed, every sample reached the plateau indicating an adequate sampling depth. The α-diversity of intestinal microbiota was analyzed using the indices of Shannon, Simpson, ACE, and Chao 1. The result in Table 8. The β-diversity analysis was performed to compare the overall microbial profiles and obtain the results shown in Supplementary Figure S3 without a notable difference (*P* > 0.05).

**Table 8:** Effects of GOD-supplemented feed on the cecal α-diversity of broilers.

**Variable**	**Ctr**	**Ant**	**GOD300**	**GOD600**	**GOD1200**	**SEM**	***P*-value**
Shannon index	4.12	3.95	4.06	4.22	4.15	0.051	0.645
Simpson index	0.06	0.07	0.06	0.05	0.05	0.012	0.677
ACE index	479.84	480.83	488.62	485.16	491.68	5.485	0.953
Chao 1 index	487.36	484.55	497.91	490.66	498.19	5.772	0.899

**Figure 3:**
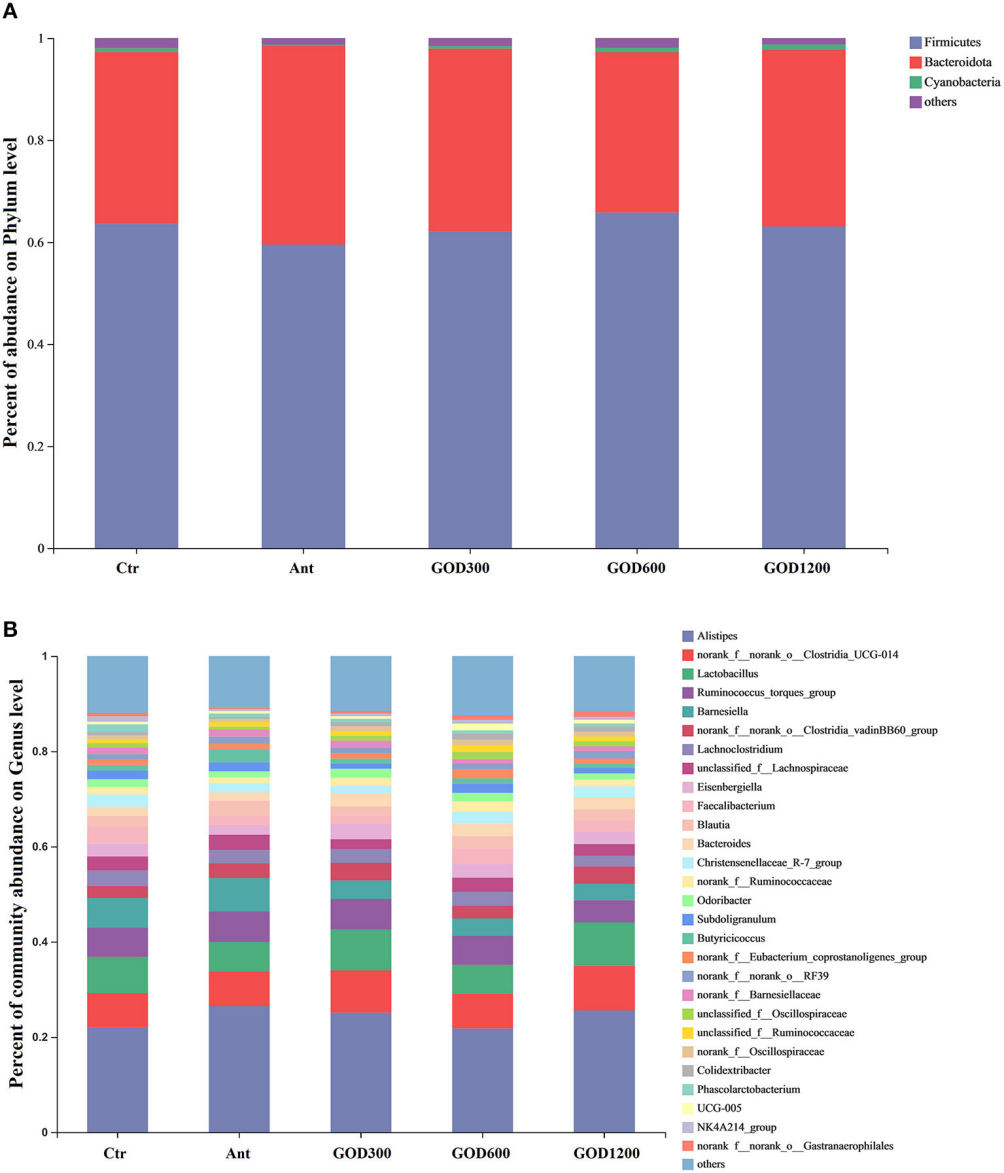
Compositions of cecal microbiota identified under different concentrations of GOD and the antibiotic-supplied diet on day 42 of the broilers at phylum **(A)** and genus levels **(B)** (*n* = 6, *P* < 0.05). Ctr, negative control fed with the basal diets; Ant, positive control fed with the basal diets added 50 mg/kg aureomycin; GOD300, GOD600, and GOD1200, the basal diets supplied with 300, 600, and 1,200 U/kg glucose oxidase, respectively. Ctr, negative control group fed with the basal diets; Ant, positive control group fed with the basal diets added 50 mg/kg aureomycin.

**Figure 4:**
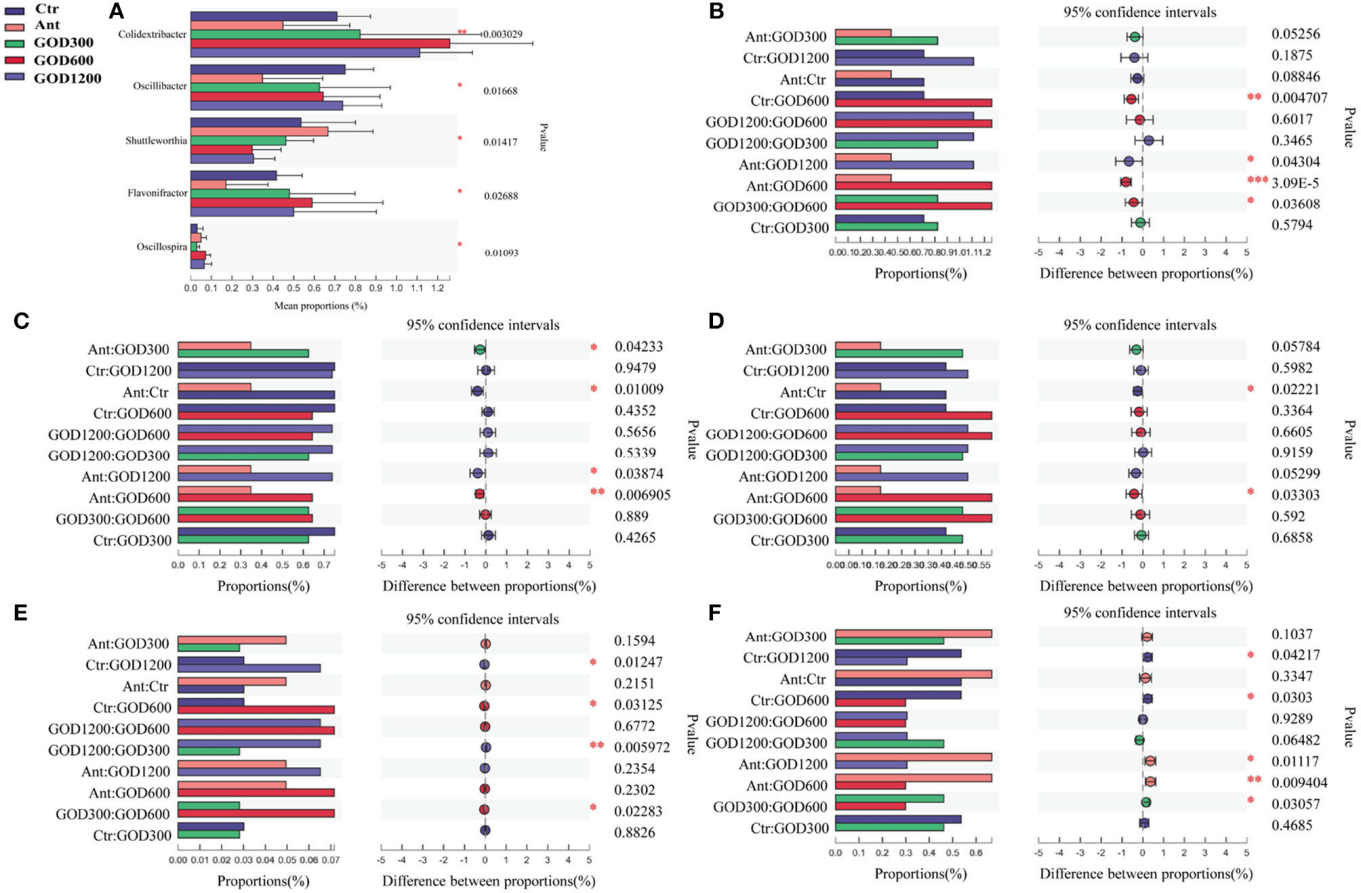
Five significantly differential bacteria (*P* < 0.05) of the cecal microbiota under different concentrations of GOD and the antibiotic-supplied diet on day 42 of the broilers at the genus level. (*n* = 6, *P* < 0.05) among multiple comparisons **(A)** and pairwise comparisons for each one **(B–F)**. **(B)** for *Colidextribacter*, **(C)** for *Oscillibacter*, **(D)** for *Flavonifractor*, **(E)** for *Oscillospira*, and **(F)** for *Shuttleworthia*. Ctr, negative control fed with the basal diets; Ant, positive control fed with the basal diets added 50 mg/kg aureomycin; GOD300, GOD600, and GOD1200, the basal diets supplied with 300, 600, and 1,200 U/kg glucose oxidase, respectively. Ctr, negative control group fed with the basal diets; Ant, positive control group fed with the basal diets added 50 mg/kg aureomycin.

To assess the role of the GOD in feed, the taxonomic compositions of cecal microbes were compared at phyla and genus levels among the treatments (Figure 3). At the phylum level, it could be concluded from the relative abundance maps that Firmicutes, Bacteroidota, and Cyanobacteria are the main phyla in the cecal bacterial community of broilers. Firmicutes constituted nearly 63.32% of the whole sequences followed by ~35% Bacteroidota and ~0.68% Cyanobacteria. When compared to the positive control group, broiler-fed GOD had a higher relative abundance of Firmicutes and a lower relative abundance of Bacteroidetes. At the genus level, the distribution shown in Figure 3B was summarized to show that 28 dominant genera (*Alistipes, g_norank_f_norank_o_Clostridia_UCG-014, Lacto bacillus, Ruminococus_torques_group, Barnesiella, norank_f_norank_o_Clostridia_vadinBB60_group, Lachnoclostridium, unclassified_f_Lachnospiraceae, Eisenbergiella, Faecalibacterium, Blautia, Bacteroides, Christensenellaceae_R-7_group, norank_f_Ruminococcaceae, Odoribacter, Subdoligranulum, Butyricicoccus, norank_f_Eubacterium_coprostanoligenes_group, norank_f_norank_o_RF39, norank_f_Barnesiellaceae, unclassified_f_Oscillospiraceae, unclassified_f_Ruminococcaceae, norank_f_Oscillospiraceae, Colidextribacter, Phascolarctobacterium, UCG-005, NK4A214_group*, and *norank_f_norank_o_Gastranaerophilales*.) changed among these groups after various supplementations were added to the basal diet. Specifically, after performing the Kruskal–Wallis H test, five significantly differential bacteria (*P* < 0.05) were noticed at the genus level in the multiple-group comparisons test (Figure 4A). They were *Colidextribacter, Oscillibacter, Shuttleworthia, Flavonifractor*, and *Oscillospira*. Subsequently, the Wilcoxon rank-sum tests were employed for each of the five bacteria to implement a pairwise comparison, and the results can be seen in Figures 4B–F. Compared with the basal diet group, GOD600 and GOD1200 showed a significant increase in the relative abundance of *Oscillospira* (*P* < 0.05). The abundance of *Colidextribacter* was enriched significantly in GOD600 as well (*P* < 0.01). More and varying degrees of effects were noticed when GOD-supplied groups were compared with Ant. All three GOD-supplemented diets caused a significant increase in the relative abundance (*P* < 0.05) of *Oscillibacter*. GOD600 produced significant (*P* < 0.05) and even extreme differences (*P* < 0.01) in the genera of *Flavonifractor* and *Colidextribacter*, respectively. A noticeable increase in *Colidextribacter* also took place in GOD1200 in broilers' cecal microbiota (*P* < 0.05). However, in contrast to the aforementioned conclusions, we noticed that *Shuttleworthia* exhibited different trends in the other four genera. The control group (negative and positive types) brought about a significant increase (*P* < 0.05) in this genus regardless of whether it was compared with GOD600 or GOD1200. There was an extreme increase in Ant when compared with GOD600 (*P* < 0.01). Moreover, GOD300, which exerted a few impacts on the other four genera, significantly raised the content of this genus compared with GOD600 (*P* < 0.05). Additionally, except for the comparisons of control groups and the additive-supplied groups, we also noticed that the abundance of *Flavonifractor* and *Oscillibacter* genera in the basal diet group was significantly higher than that in the antibiotic control group.

### Functional potential of cecal microbiota composition

**Figure 5:**
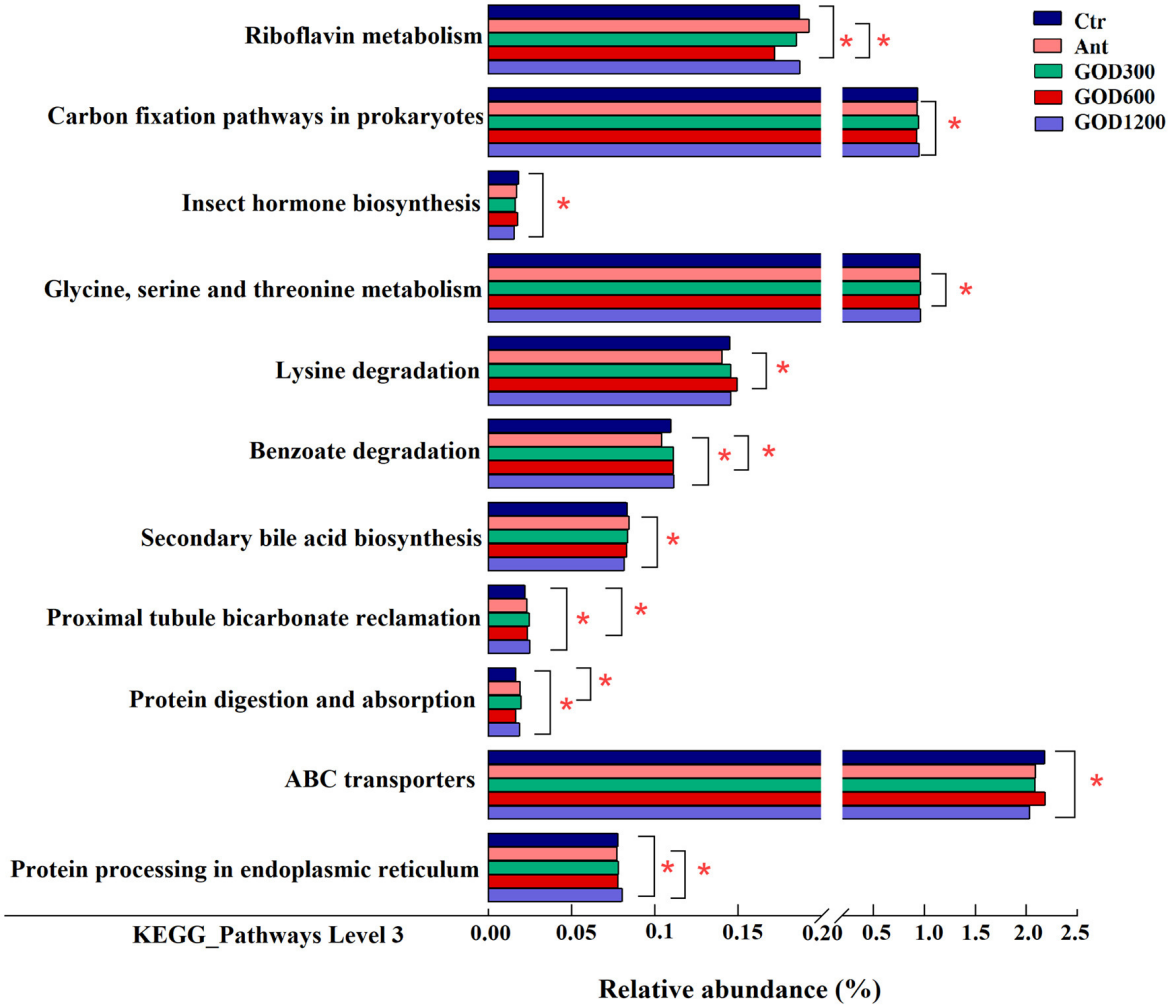
The mean proportion in predicted pathways grouped into level 3 functional categories of the cecal microbiota in various treatments on day 42 (*n* = 6, *P* < 0.05). Ctr, negative control fed with the basal diets; Ant, positive control fed with the basal diets added 50 mg/kg aureomycin; GOD300, GOD600, and GOD1200, the basal diets supplied with 300, 600, and 1,200 U/kg glucose oxidase, respectively. ^*^Means there are significant differences in the enrichment of this pathway between the two groups. Ctr, negative control group fed with the basal diets; Ant, positive control group fed with the basal diets added 50 mg/kg aureomycin.

All alterations in the presumptive function were evaluated using PICRUSt2 in the cecal microbiota at 42 days of the broilers. There were 43 pathways predicted at level 2 of the KEGG pathways. However, no significant differences were found among them, and specific information was not shown here. Furthermore, it was found that some pathways were differentially enriched at level 3 among groups, and 289 KEGG categories were identified in total. We conducted six pairwise comparisons between each of the control groups and each concentration of the GOD-supplied groups, that is, Ctr vs. GOD300, Ctr vs. GOD600, Ctr vs. GOD1200, and Ant vs. GOD300, Ant vs. GOD600, Ant vs. GOD1200, respectively. The results revealed in Figure 5 illustrated that, compared with the basal diet (i.e., negative control group), the GOD treatments significantly influenced the abundance of six functional pathways. Meanwhile, the abundance shifted remarkably in seven functional pathways when this comparison was made between the antibiotic-supplied group (i.e., positive control group) and the GOD treatments.

Specifically, the negative control group showed a significantly larger abundance of “Riboflavin metabolism” and “Proximal tubule bicarbonate reclamation” pathways against GOD600 (*P* < 0.05). As for the “ABC transporters” and “Insect hormone biosynthesis” pathways, GOD1200 exerted a significant influence on making them decrease (*P* < 0.05). In addition, the pathways of “Proximal tubule bicarbonate reclamation,” “Protein digestion and absorption,” and “Protein processing in endoplasmic reticulum” were all differentially enriched in GOD treatment groups (*P* < 0.05). After the comparison between positive control and GOD groups, we observed that the former had a significantly lower abundance of several functional pathways (*P* < 0.05), including “Carbon fixation pathways in prokaryotes,” “Glycine, serine and threonine metabolism,” “Lysine degradation,” “Benzoate degradation,” and “Protein processing in endoplasmic reticulum.” Whereas, a significantly lower abundance of “Secondary bile acid biosynthesis” and “Riboflavin metabolism” existed in GOD (*P* < 0.05). The details about how different concentrations of GOD interacted with both control groups are shown in Figure 5.

## Discussion

### Growth performance, immune organ indexes, and serum test results

Based on the aforementioned results, the antibiotic group showcased a relatively higher feed intake and a low weight gain against GOD1200 during the stage of starting phase (1–21 days). A few research types showed this tendency of the antibiotic (Khan et al., 2012; Elagib et al., 2013). However, it implied that the GOD1200 supplement could relatively improve the feed conversion efficiency. For the same index, GOD600 and GOD1200 exerted a better efficiency than the basal diet group during the growing phase (22–42 days). These could serve as evidence for GOD in the role of a growth promoter. The F:G ratio were significantly affected by the dietary GOD supplied, especially in GOD1200, indicating that the dietary GOD-supplied could help cut costs and increase farming efficiency. Nevertheless, no other differences in growth indexes were observed, and we inferred that there were specific conditions that caused the disparity. A better feeding environment, various dosages of additives as well as the broiler's breed could result in this disparity (Hashemipour et al., 2013; Ahiwe et al., 2021). The unchanged relative weights of immune organs implied that additives or antibiotics may act to cause no significant shift in the immune organs under certain circumstances. It can be further conjectured that the three levels of GOD have no adverse effect on the development of the immune organ of the white-feathered broilers during their growth status.

Except for the relative weights of organs, some serum tests, such as biochemical, cytokines, and antioxidant indexes, could reflect the condition of the immune cells and the health level of the host medically as well. Several indexes changed significantly in the present study. A significant reduction in ALP, whose activity in blood was considered an essential indicator for assessing the adequacy of phosphorus (Li et al., 2020), was found in GOD300 on day 21 compared with the control group. Some experiments showed that the lower activity of ALP stood for a healthy broiler status (Skalicka et al., 2000), whereas we cannot draw a conclusion from this index alone. The immunity indicator, TGF-β cytokine, significantly decreased when GOD (GOD600 or GOD1200) was compared with the basal diet group. Cytokines are mainly synthesized and secreted from various immune cells in the form of polypeptides or glycoproteins. Their existence can mediate the interaction between cells that perform various biological functions (Haddad, 2002). As a member of them, TGF-β is regarded as a considerable enforcer of immune homeostasis and tolerance, especially for regulating inflammatory processes (Batlle and Massagué, 2019; Fasina and Lillehoj, 2019) with a dual effect (Pickup et al., 2017; David and Massagué, 2018). The broiler growth stage of 1–21 days is typically associated with an immature immune system (Song et al., 2021). Based on the significant TGF-β difference on day 21, we inferred that GOD- and Ant-supplied diets gave broilers a better ability toward stress stimulation at a specifically tested concentration. It could be further verified by the remarkable MDA increase and the SOD decrease in the basal diet group at 21 days, which was consistent with the former reports (Wang et al., 2018).

With the characteristic of high lipid content, broilers are easily induced to produce ROS (Bai et al., 2017). As for the results of this study, a higher content of MDA in Ctr represented attenuated antioxidant protection when the ROS increased (Yang et al., 2008). Correspondingly, SOD and GSH-Px, the two enzymes, were used to remove excess ROS (Ko et al., 2004) and showed a significant positive advantage in GOD1200. Meanwhile, the high GOD supplement exerted a similar and even better effect in contrast to the aureomycin-added diet on days 21 and 42, respectively. The changes in antioxidant parameters illustrated that the addition of GOD could reduce lipid peroxidation for broilers to a certain extent. In animals, the endogenous antioxidant defense system and immune system rely on external sources (Pamplona and Costantini, 2011). Both systems can facilitate the development of a robust antioxidant capacity. Herein, our results initially identified that the GOD could improve broilers' immune and antioxidant capacity, thereby further improving their health status.

### Immunohistochemical result and the gene expression of tight junctions in the jejunum

The gut is the most significant immune organ undertaking both the tolerance to dietary antigens and the immune defense for broilers. The organized gut-associated lymphoid tissues (GALT) in birds generate efficient responses with secretory IgA (Fagarasan et al., 2009), known as SIgA, to maintain mucosal homeostasis (Lammers et al., 2010; Curtis, 2017). For this experiment, the SIgA content showed a conspicuous decrease in the aureomycin-added group compared with all other groups. The reduction in SIgA could influence the pro-inflammatory downregulation ability of the host (Boullier et al., 2009), indicating that, after 42 days of feeding, the antibiotic did not give an advantage to the immunologic barrier. The results proved that GOD might be an advantageous alternative to antibiotics. Alongside the immunologic barrier, the mechanical barrier is the other primary component of intestinal mucosal immunity (Reynolds et al., 1996). Some studies reported that the intestinal mechanical barrier of chickens could be affected by the factor of dietary components' alteration (Fasina et al., 2006; Ma et al., 2021). In this study, the high-level GOD supplement increased the relative abundance of *MUC-2* and *ZO-1*. These two are the major components of adherence junctions (AJs) and tight junctions (TJs), respectively, which act as the crucial parts of the physical gut barrier (Ballard et al., 1995; Anderson et al., 2012). It was reported that *MUC-2* exerted a major role in protecting the intestinal epithelium in preventing infection and maintaining the integrity of the intestinal mucosal barrier (McGuckin et al., 2011). As a member of the tight junctions, the *ZO-1* is negatively correlated with intestinal permeability (Alhotan et al., 2021). The results of this study indicated that the additive supplied could enhance the intestinal physical barrier of broilers. This inference can also be supported by the D-Lac's change in serum in this study, as the D-Lac is used to detect intestinal permeability and is an indirect indicator of the intestinal barrier (Fukudome et al., 2014; Wang J. et al., 2022). Taken together, GOD at a certain concentration can facilitate the integrity of the intestinal epithelium and enhance the mucosal immune capacity of broilers, thereby promoting their healthier living conditions.

### Intestinal microbiota comparison in the cecal contents and the functional prediction result

As the chief functional part in the distal intestine, the cecum has received increasing attention for its importance in chicken metabolism since it contains a vast majority of gut bacteria and has a significant fermentation ability with a lower passage rate (Pourabedin and Zhao, 2015). The cecal microbiota of chickens can influence the host health and productivity by regulating nutrient absorption and metabolism, immune response, and pathogen invasion (Stanley et al., 2014; Huang et al., 2018). There are a few reports on the main site of GOD's action in the intestinal segments. To the best of our knowledge, these reports include its biochemical features of oxygen consumption, gluconic acid production, and negative effects on certain pathogenic bacteria in broilers' gut (Liang et al., 2022). Therefore, to better understand and complement the current mechanism of GOD supplement in broilers and to evaluate its application effects at different concentrations, shifts in the cecal microbiota among five groups were observed in this study.

First, we noticed that the results of the data analysis for α-diversity were not statistically significant after a 42-day feeding. These results are similar to the intestinal diversity results reported after certain additives were added to the feed (Ma et al., 2021; Liu C. et al., 2022). However, it is possible that rare numbers found in a small population could make a significant difference for the host (Shang et al., 2018). Therefore, the sequences were further analyzed at phylum and genus levels to identify the cecal differential bacteria. Similar to previous studies, Firmicutes, Bacteroidota, and Cyanobacteria are the dominant phyla in the broiler cecal bacterial community (Mohd Shaufi et al., 2015; Dai et al., 2021; Segura-Wang et al., 2021).

At the genus level, *Colidextribacter, Oscillibacter, Flavonifractor, Oscillospira*, and *Shuttleworthia* emerged after multiple comparisons and were identified as biomarkers to distinguish the groups for whether supplied with GOD. The former four genera showed a significant increase in GOD600 and GOD1200 compared with control groups. The first one, *Colidextribacter*, has been reported to promote the production of short-chain fatty acids (SCFAs) (Oakley et al., 2014; Wang Q. et al., 2022) and inosine (Lee et al., 2020; Guo et al., 2021). Studies on chickens illustrated that SCFAs can reduce inflammation in the intestines (Wu et al., 2016). For example, butyrate can repress cell invasion in pathogenicity island caused by *Salmonella*, a pathogen of concern to the global poultry industry (Gantois et al., 2006), and the microbiota-derived butyrate could also effectively ameliorate certain immune system diseases (He et al., 2020). Correspondingly, substantial evidence highlighted that inosine has broad anti-inflammatory and immunomodulatory properties (Haskó et al., 2000, 2004; da Rocha Lapa et al., 2012). The second noticed genus, *Oscillibacter*, whose clade was regarded as a potential n-butyrate producer (Gophna et al., 2017; Contreras-Dávila et al., 2021), was placed in the family *Ruminococcaceae*. In recent studies, this family showed a high correlation with the increase in bodyweight and tight junction protein expression for birds (Dai et al., 2021; Farkas et al., 2022). *Oscillibacter* is considered a potentially beneficial microbe as it plays a crucial role in sugar fermentation (Ze et al., 2012) and starch degradation (Kim et al., 2014) and is positively associated with feed efficiency for broilers (Liu J. et al., 2021). One of its species, *Oscillibacter ruminantium*, was found to be negatively correlated to *Salmonellac* (Pedroso et al., 2021) after the investigation of chickens' cecal content. Moreover, *Oscillibacter* was also demonstrated to reduce blood triglyceride concentration and the negative reaction to stress for research on humans (Jiang et al., 2015; Tong et al., 2020; Liu X. M. et al., 2022). *Flavonifractor* is also a butyrate-producing producer (Meng et al., 2019). It was positively correlated with ADG and could improve the growth performance of broilers (Zhang et al., 2021). Some of its clades were the key to catalyzing and initiating flavonoid metabolism. Currently, many studies on the flavonoid showed its marked effects on improving growth performance and the antioxidant capacity of broilers (Kamboh and Zhu, 2013). In light of the predominance of GOD in this study and the positive correlation between this genus and GOD in the cecum, we speculated that the combination of flavonoid and glucose oxidase might exert better anti-inflammatory, bacteriostatic, and immunity enhancement effects on animals as a novel feed additive (Wang et al., 2020; Yang G. et al., 2021). Along with *Oscillibacter*, the *Oscillospira* genus belongs to the family of *Ruminococcaceae* and is also regarded as a short-chain fatty acid butyrate producer. Accordingly, it could downregulate the expression of genes encoding pro-inflammatory cytokines and prevent inflammation in the host (Cushing et al., 2015; Gophna et al., 2017). *Ruminococcaceae* have been reported to be highly positively correlated with the gene expression of *ZO-1* (Dai et al., 2021). This can be confirmed in this study as the *Oscillibacter* and *Oscillospira* genera in GOD (GOD600 and GOD1200) were significantly higher than those in control groups, and the trend in *ZO-1* expression was the same between GOD and control groups. Moreover, based on human research, the significant *Oscillospira* decrease was associated with obesity-related chronic inflammatory and metabolic diseases (Yang J. et al., 2021). Hence, it may become a next-generation probiotic candidate for symptom relief in broilers. The genus of *Shuttleworthia* showed a noticeable increase in control groups, especially for GOD at the concentration of 600 or 1,200 U/kg. Compared with the former four genera, the tendency was the opposite. Combined with other parameters tested in the present study, although there were some benefits found in this genus for animals, we conjectured that its negative effects played a leading role in the control groups (Liu Y. et al., 2021). These negative effects might be attenuated with the addition of glucose oxidase. The genera above all belong to the anaerobic genus, which could confirm that the GOD additive is conducive to creating a better anaerobic condition for the proliferation of beneficial bacteria into the gut. In addition, gluconic acid was rarely absorbed in the small intestine and primarily fermented by specific bacteria to produce SCFAs in the cecum (Biagi et al., 2006; Huyghebaert et al., 2011), which can be further verified. Furthermore, analysis of characteristic genera also provided a profound interpretation of the phenomenon of a higher F:G index in GOD.

According to the predicted functional profiles analyzed by PICRUSt, the microorganisms of broilers' cecum were mainly enriched in functions of metabolism, organismal systems, environmental information processing, and genetic information processing, of which the pathways associated with metabolism occupied the majority. Clearer differences were observed in level 3 KEGG pathways.

The metabolic pathways, such as riboflavin metabolism, insect hormone biosynthesis, and secondary bile acid biosynthesis, displayed varying degrees of benefits for broilers. The riboflavin metabolism was reported to be related to mitochondria-mediated apoptosis, and the flavoprotein participating in this metabolism may contribute to superoxide production along with mitochondrial energy metabolism (Balasubramaniam and Yaplito-Lee, 2020; Liao et al., 2021). It implied that this metabolism might play a part in oxidative stress. In our study, GOD supplementation of 600 U/kg significantly decreased this metabolic pathway in both control groups, indicating that the broilers were found to thrive in better living conditions after the GOD supply. The enrichment in the basal diet group of “insect hormone biosynthesis” may demonstrate that GOD1200 played a role in parasite removal, which contributed to a better intestinal condition and immune capacity for broilers. Moreover, the secondary bile acid biosynthesis enriched in Ant could provide a reasonable explanation for the phenomenon that the kind of biosynthesis always occurs in specific microbiota after antibiotic therapy to fight against pathogenic bacteria (Buffie et al., 2015; Koenigsknecht et al., 2015), similar to *Clostridium difficile* (Rupnik et al., 2009; Buffie et al., 2012). Notably, there were more differences in metabolic function pathways when comparing Ant and GOD. Among them, the amino acid, the energy, and the xenobiotic biodegradation and metabolism pathways were more abundant in the cecal flora in the medium and high concentration GOD. Combined with other indexes of the advantages displayed in this study, it was speculated that the aforementioned pathways mainly took effect in promoting nutrient absorption, digestion, and resistance to external disturbances. This could also be supported by significant enrichment of “protein digestion and absorption” in GOD compared with the basal diet group.

Meanwhile, the pathway of “proximal tubule bicarbonate reclamation” was reported to play an important role in maintaining the acid–base balance in host organisms (Guo et al., 2014). Since the acid–base balance can be easily disturbed by internal and external factors, such as diet, environmental conditions, and metabolism (Anrewaju et al., 2007), we inferred that the enrichment of certain genes in response to GOD (GOD600 and GOD1200) treatment may provide broilers with increased resistance to various stresses. This is supported by our findings from the antioxidant analysis in this study.

Furthermore, two pathways involved in information processing drew our attention: “protein processing in endoplasmic reticulum” and “ABC transporters.” Previous research showed that the pathway of “protein processing in endoplasmic reticulum” can have different effects on chickens depending on the conditions they are exposed to. For example, the pathway might be enriched and participate in the apoptotic process in response to toxic substances (Sun et al., 2021) or environmental changes (Srikanth et al., 2019), however, it can also be downregulated in response to the infection by parasites (Li et al., 2019). A complicated process must exist in a broiler's body in response to various conditions. The results of our study suggest that this pathway may have a positive effect on broilers treated with GOD1200, even though further research is needed for validation. Similarly, the “ABC transporters” accounting for a high abundance in the negative control group have been shown to play a role in both multidrug resistance and nutrition uptake (Rice et al., 2014; Hofmann et al., 2019). Based on the results aforementioned, we inferred that the 1,200 U/kg GOD-supplied group may help to reduce the microbial resistance in contrast to the control group.

Overall, evidence for all the pathways illustrated that specific GOD supplementations may have a beneficial effect on the health of birds, particularly in GOD1200, whereas the antibiotic additive may not. Even though the predictive tool of PICRUSt is well-used, it cannot confirm the functional capabilities of the metagenome with absolute certainty. Herein, further research is needed to validate our findings.

## Conclusion

In conclusion, our research showed that a diet supplemented with GOD resulted in a higher feed conversion efficiency and enhanced the internal body environment of broilers, and the concentration of 1,200 U/kg could be the recommended dosage based on the overall results. Unlike AGPs, which can disrupt the integrity of small intestinal epithelium and microbiota, GOD provides a non-pharmacological manner for strengthening the immunologic barrier and maintaining a healthy intestinal microecology. These findings deepen our understanding of the potential benefits of GOD as a feed additive and highlight its potential as a safe and effective substitute for AGPs as a growth promoter in poultry production.

## Data availability statement

The datasets presented in this study can be found in online repositories. The names of the repository/repositories and accession number(s) can be found in the article/Supplementary material.

## Ethics statement

The animal study was reviewed and approved by Animal Care and Use Committee of the Feed Research Institute of the Chinese Academy of Agricultural Science.

## Author contributions

WZ, RW, and ZX conceived and designed the experiments. WZ, YH, and NC performed the animal experiments. WZ analyzed the data and wrote the manuscript. XS supervised and provided continuous guidance for the experiments. All authors discussed the results and reviewed the manuscript. All authors contributed to the article and approved the submitted version.
