# Supplementary material for: Glucose oxidase as an alternative to antibiotic growth promoters improves the immunity function, antioxidative status, and cecal microbiota environment in white-feathered broilers
Source: Front Microbiol. 2023 Mar 3;14:1100465. doi: 10.3389/fmicb.2023.1100465 (PMC10020722; doi:10.3389/fmicb.2023.1100465)
Supplement: Supplementary file 1 [file Data_Sheet_1.docx]

Supplementary Materials

The Glucose Oxidase as an Alternative for Antibiotic Growth Promoters Improves the Immunity Function, Antioxidative Status, and Cecal Microbiota Environment in White-feathered Broilers

**Wenyu Zhao^1^, Yuan Huang^1^, Na Cui^1^, Ruiguo Wang^1^, Zhiming Xiao^1^, and Xiaoou Su^1^***

^1^Key Laboratory of Agro-Product Quality and Safety of the Ministry of Agriculture, Institute of Quality Standards and Testing Technology for Agro-Products, Chinese Academy of Agricultural Sciences, NO. 12 Zhong-guan-cun South Street, Haidian District, Beijing 100081, People's Republic of China

*** Correspondence:**Corresponding Author
suxiaoou@caas.cn


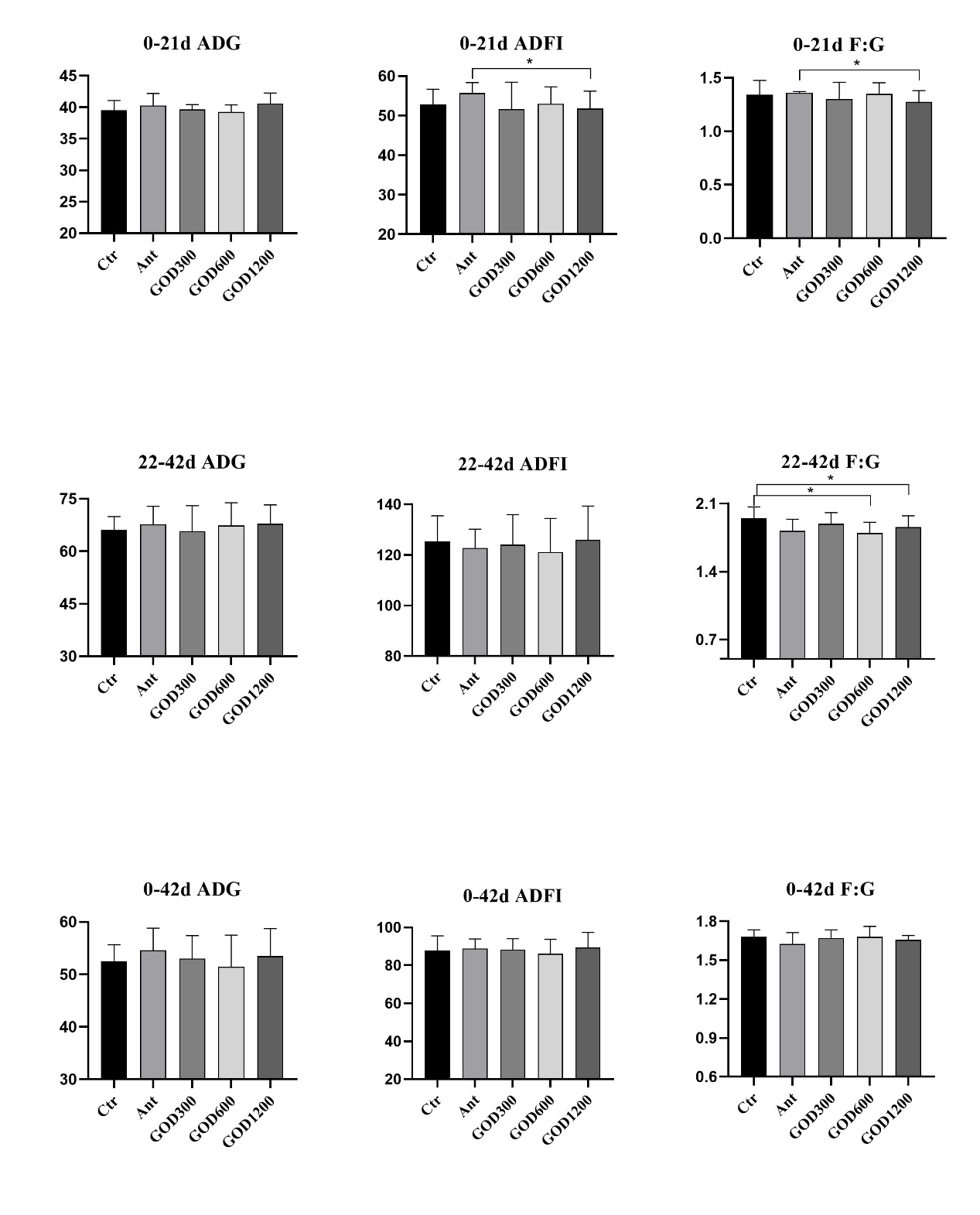


**Figure S1.** The t-test results of the effects GOD acted on the growth performance of broilers during different phases. Ctr: negative control fed with the basal diets; Ant: positive control fed with the basal diets added 50 mg/kg aureomycin; GOD300, GOD600, GOD1200: the basal diets supplied with 300 U/kg, 600 U/kg and 1200 U/kg glucose oxidase, respectively. **P* < 0.05, compared between the two groups.

**
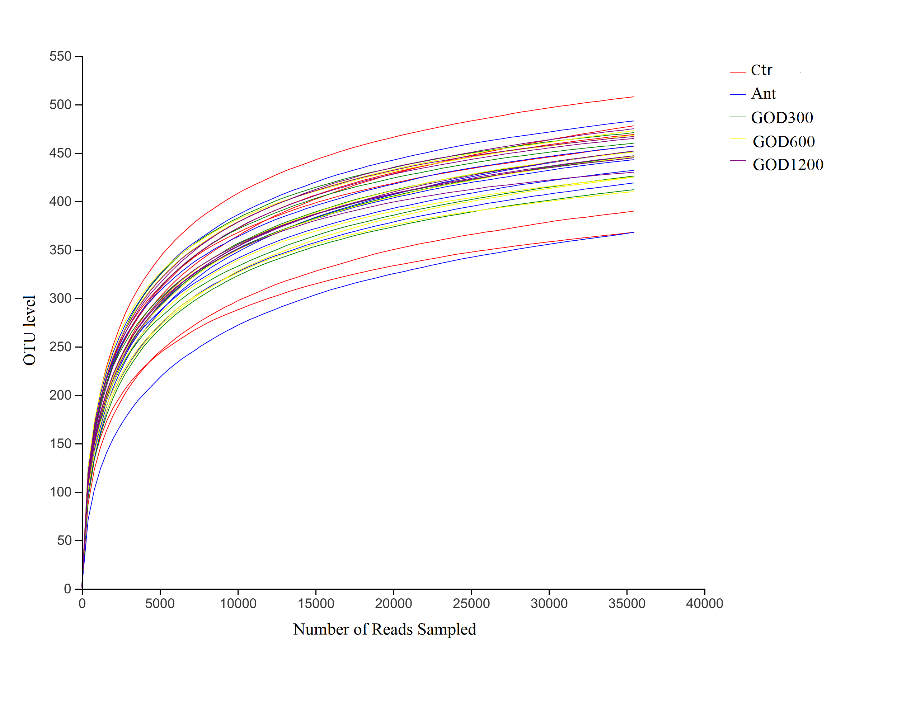
Figure S2.** Effects of dietary supplementation with GOD on the rarefaction curve of broilers on day 42. Ctr: negative control fed with the basal diets; Ant: positive control fed with the basal diets added 50 mg/kg aureomycin; GOD300, GOD600, GOD1200: the basal diets supplied with 300 U/kg, 600 U/kg and 1200 U/kg glucose oxidase, respectively.


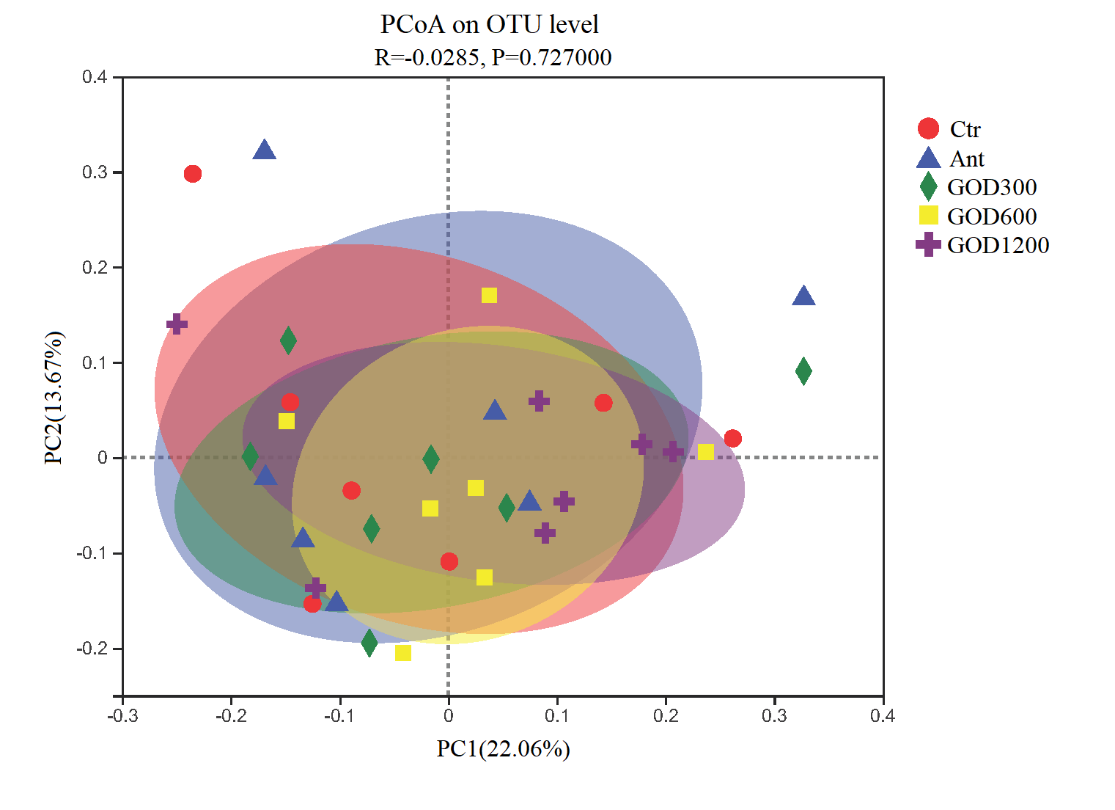
**Figure S3.** Principal coordinate analysis (PCoA) of dietary supplementation with GOD on the cecal microbial β-diversity of broilers on day 42. Ctr: negative control fed with the basal diets; Ant: positive control fed with the basal diets added 50 mg/kg aureomycin; GOD300, GOD600, GOD1200: the basal diets supplied with 300 U/kg, 600 U/kg and 1200 U/kg glucose oxidase, respectively.
